# Facile fabrication of wire-type indium gallium zinc oxide thin-film transistors applicable to ultrasensitive flexible sensors

**DOI:** 10.1038/s41598-018-23892-4

**Published:** 2018-04-03

**Authors:** Yeong-gyu Kim, Young Jun Tak, Hee Jun Kim, Won-Gi Kim, Hyukjoon Yoo, Hyun Jae Kim

**Affiliations:** School of Electrical and Electronic Engineering, Yonsei University, 50 Yonsei-ro, Seodaemun-gu, Seoul, 03722 Republic of Korea

## Abstract

We fabricated wire-type indium gallium zinc oxide (IGZO) thin-film transistors (TFTs) using a self-formed cracked template based on a lift-off process. The electrical characteristics of wire-type IGZO TFTs could be controlled by changing the width and density of IGZO wires through varying the coating conditions of template solution or multi-stacking additional layers. The fabricated wire-type devices were applied to sensors after functionalizing the surface. The wire-type pH sensor showed a sensitivity of 45.4 mV/pH, and this value was an improved sensitivity compared with that of the film-type device (27.6 mV/pH). Similarly, when the wire-type device was used as a glucose sensor, it showed more variation in electrical characteristics than the film-type device. The improved sensing properties resulted from the large surface area of the wire-type device compared with that of the film-type device. In addition, we fabricated wire-type IGZO TFTs on flexible substrates and confirmed that such structures were very resistant to mechanical stresses at a bending radius of 10 mm.

## Introduction

Medical science and biotechnology seek to improve health and extend longevity^1,2^. Biosensors constitute a new paradigm in medical technology. Biosensors detect biological elements, affording prompt and accurate diagnoses at low cost^3,4^. Especially, field-effect transistor (FET)-based biosensors have been intensively investigated because they afford the advantages of direct transduction and high sensitivity at a low driving voltage^5,6^.

Meanwhile, in recent years, non-invasive biosensors are used to measure biomaterials in sweat, saliva, and tears instead of blood^7,8^. Such biosensors must be highly sensitive; the concentrations of biomaterials in sweat, saliva, and tears are lower than in blood. Many studies employed low-dimensional nanomaterials such as quantum dots, carbon nanotubes (CNTs), graphene, and MoS_2_ to improve biosensor sensitivity^9–12^. However, fabrication of such materials is expensive and it is difficult to fabricate uniform films.

In this study, we fabricated indium gallium zinc oxide (IGZO) in a shape of networked nanowires to improve the sensitivity. Oxide semiconductors have attracted considerable attention substitutes for silicon in various applications because of its low power consumption, high uniformity for large area fabrication, high transparency in visible region, and diversity of deposition methods including solution process^13–15^. Although several studies have reported fabrication of IGZO wires, they employed complex processes, such as laser irradiation and photolithography or the fabricated wires were hard to apply in thin-film transistor (TFT) applications^16–19^. Recently, TFTs using perforated IGZO films as active layers have been applied to sensing applications^20^. However, complicated processes, such as reactive ion etching (RIE), were used to control the template formation; such methods are expensive and difficult. The method that we develop here allows simple fabrication of networked nanowires using a self-formed cracked template, and does not require complex processes, unlike previous studies. A cracked template was readily formed by coating the substrate with a template solution followed by drying without any additional treatment; we thus fabricated highly interconnected wire structures using this template. Such interconnected IGZO nanowires were used as the active layers of TFTs; the electrical characteristics of wire-type IGZO TFTs were controlled by varying the width and density of the wires. These wire-type IGZO TFTs were used to sense glucose solution and the sensitivity of wire- and film-type sensors were analyzed. Finally, wire-type IGZO TFTs were fabricated on a flexible substrate and mechanical stability of them was investigated.

## Results and Discussion

**Figure 1:**
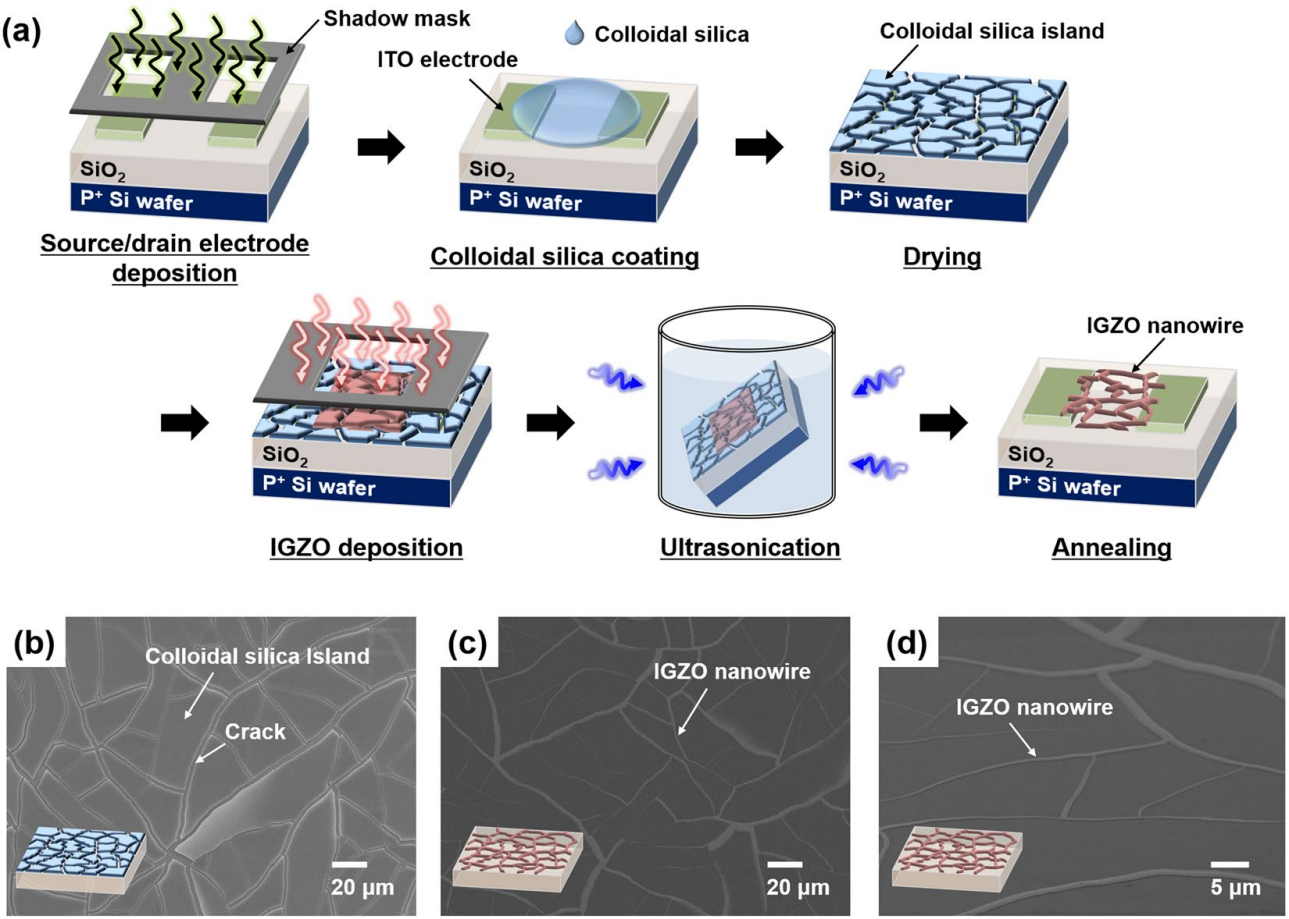
(**a**) Schematic diagram of the fabrication of wire-type indium gallium zinc oxide (IGZO) thin-film transistors (TFTs). (**b**) Surface scanning electron microscopy (SEM) image of the fabricated cracked template prepared using colloidal silica. (**c**) Surface and (**d**) tilted (≈30°) SEM images of fabricated IGZO nanowires.

The fabrication process is shown in Fig. 1a. Networked IGZO nanowires may be formed by simply coating a substrate with colloidal silica (template solution), drying it, depositing the IGZO film, and removing the template by ultrasonication in water. A cracked template is easily formed by coating the substrate with colloidal silica followed by drying as shown in Fig. 1b; no additional treatment is needed. Islands of cracked template were self-formed by agglomeration of silica nanoparticles during solvent evaporation. A cross-sectional scanning electron microscopy (SEM) image of the template after drying is shown in Figure S1a; the thickness of the layer was about 800 nm. The islands consist of physically agglomerated silica nanoparticles that are readily removed by physical treatment like ultrasonication. Figure S1b shows surface SEM images of the template before and after ultrasonication, confirming that the template can be removed by ultrasonication. Thus, by depositing an IGZO layer onto the cracked template and then removing the template, IGZO layers on island structures are removed together with the template; only IGZO layers deposited on cracks remain. As a result, a random network of IGZO wires is fabricated corresponding to cracks in the template (see Fig. 1c). Figure 1d shows a tilted (≈30°) SEM image of IGZO nanowires on the Si wafer substrate.

**Figure 2:**
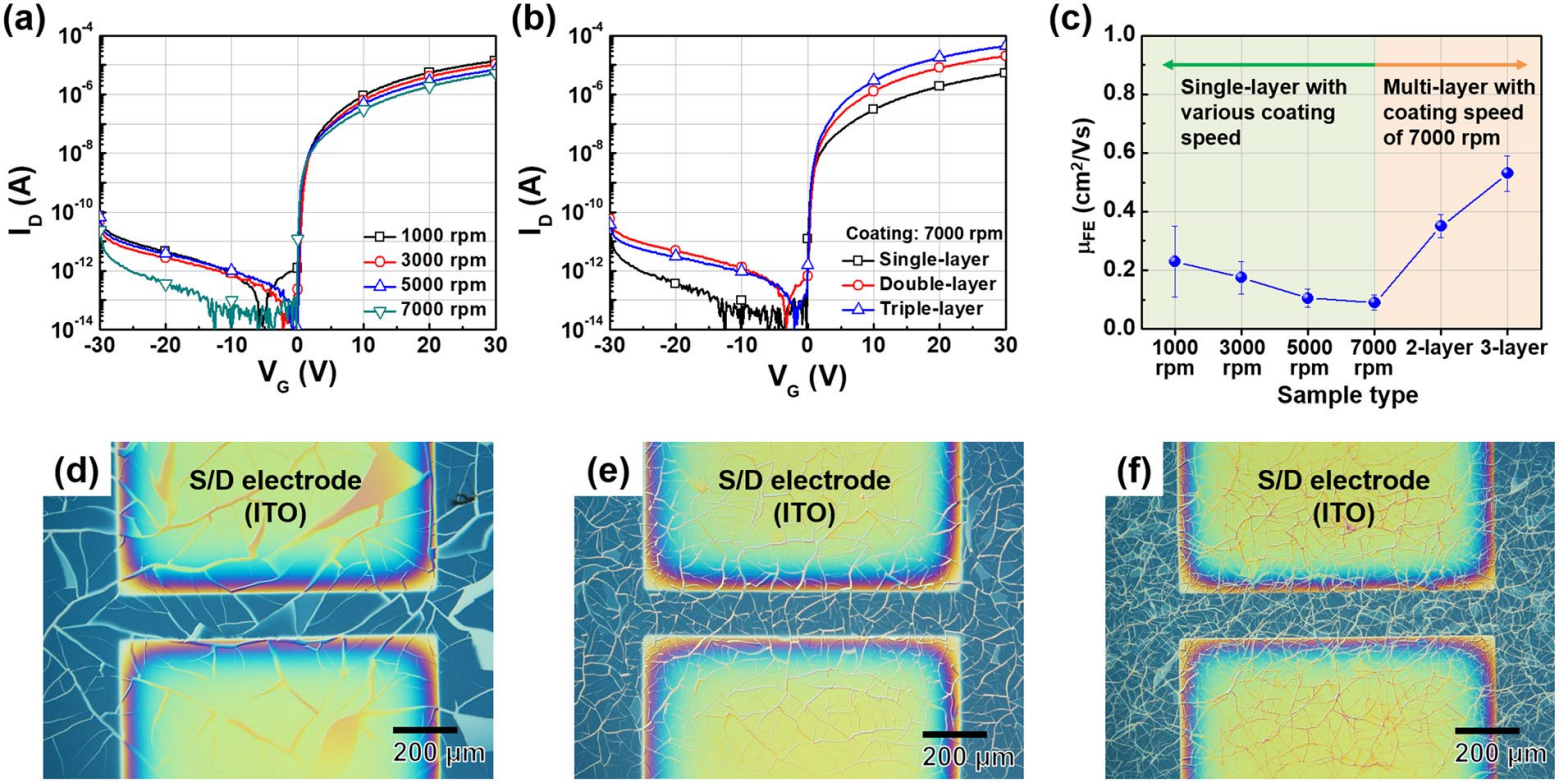
Transfer characteristics of wire-type IGZO TFTs prepared (**a**) using various spin-coating speeds for template fabrication and (**b**) using various numbers of stacked layers. (**c**) The extracted field effect mobilities of various wire-type IGZO TFTs. Optical microscopy (OM) images of single-layered wire-type IGZO TFTs prepared at spin-coating speeds of (**d**) 1,000 rpm and (**e**) 7,000 rpm, and (**f**) a triple-layered wire-type IGZO TFT prepared at a spin-coating speed of 7,000 rpm.

These IGZO nanowires were used as active layers when fabricating wire-type IGZO TFTs. Their electrical characteristics were controlled by varying the speed of template solution spin-coating as shown in Fig. 2a. The electrical characteristics of wire-type IGZO TFTs varied by the width and density of the IGZO nanowires. Figure 2d and e show optical microscopy (OM) images of wire-type IGZO TFTs prepared using spin-coating speeds of 1,000 and 7,000 rpm, respectively. When the spin-coating speed was low, silica nanoparticles in the template solution agglomerated with farther nanoparticles because the coated solution was thick^21,22^. Therefore, the template had larger islands and wider cracks compared with those of a template prepared using a high spin-coating speed. As a result, these larger islands and wider cracks at low spin-coating speed resulted in sparser and wider IGZO wires. Conversely, narrow and dense IGZO nanowires were fabricated when a high spin-coating speed was used. The wire-type IGZO TFTs prepared using a low spin-coating speed were more conductive because the net IGZO area is greater than that prepared using a high spin-coating speed. Similar results were found in previous studies^23,24^. In addition, by stacking additional layers, the electrical characteristics of wire-type IGZO TFTs could be modified, as shown in Fig. 2b. OM images of TFTs featuring single- and triple-layered IGZO nanowires prepared at a spin-coating speed of 7,000 rpm are shown in Fig. 2e and f. As the number of stacked layers increases, denser and more interconnected wire structures are obtained. Thus, the on-current increased as the number of stacked layers increased.

The field effect mobility (μ_FE_) values of IGZO TFTs were calculated as described in the Methods session and are summarized in Fig. 2c. For accurate calculations, the ratio of width and length of the TFT channels should be clearly identified. However, in wire-type IGZO TFTs, the IGZO wires are randomly distributed in the region between the source and drain (S/D) electrodes; it is thus difficult to estimate net width and length of the channel. Therefore, for μ_FE_ calculations, we used the geometrical width and length of channel (the region between the S/D electrodes). These were the maximum value of net width and the minimum value of net length, respectively; thus yielding underestimations of μ_FE_^25^. In other words, as the net area occupied by IGZO nanowires increases, the extracted μ_FE_ increases because the differences between the geometrical and net width/length decrease. Therefore, as the spin-coating speed decreases or the number of stacked layers increases, the net area covered by the IGZO nanowires increases, resulting in higher extracted μ_FE_. Device uniformity improves as the spin-coating speed increases as shown in Fig. 2c; this is why all IGZO nanowires for multi-layered structures and sensing applications were fabricated at a spin-coating speed of 7,000 rpm. Meanwhile, degradation of the electrical characteristics of conventional nanowire-based devices fabricated by deposition of pre-synthesized nanowires is inevitable problem due to the existence of contact resistance at the junctions between the pre-synthesized nanowires^26,27^. However, the wire-type IGZO TFTs fabricated in the present study were little affected by contact resistance because each wire-type IGZO layer was fabricated via one-step deposition of IGZO, minimizing the number of junctions.

**Figure 3:**
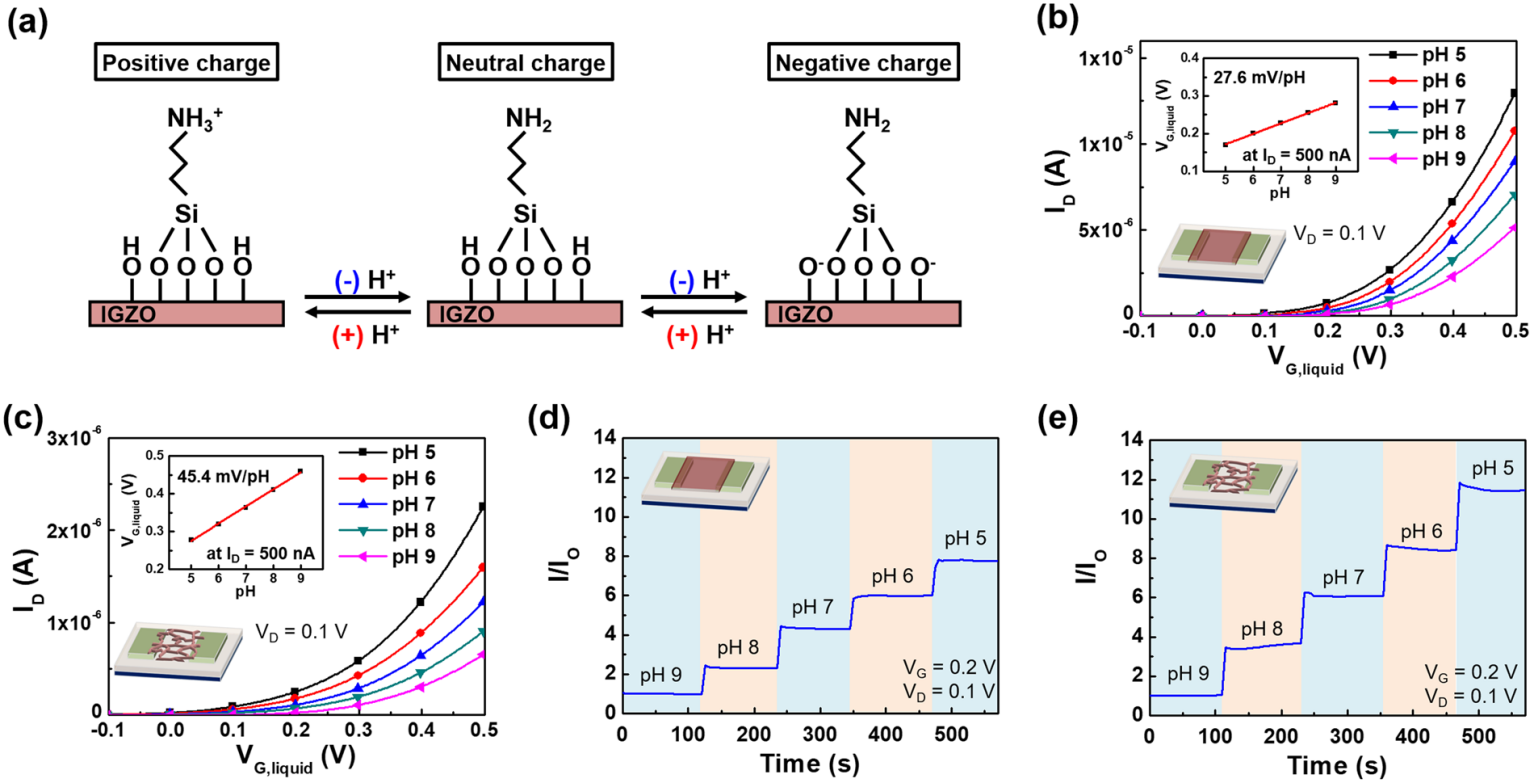
(**a**) Schematic illustration of changes in the surface charge state of APTES-treated IGZO thin-films according to the variation of pH. Electrical characteristic with various pH solutions of (**b**) film-type and (**c**) wire-type IGZO sensors. Insets in **b** and **c** shows the value of gate voltage at the drain current of 500 nA under various pH conditions. Real-time response of the drain current of (**d**) film-type and (**e**) wire-type IGZO sensors for the variation of pHs from 9 to 5.

We used the fabricated wire-type IGZO TFTs for sensing applications and compared the results with those of film-type sensors. In the case of wire-type sensor, triple-layered devices fabricated with the spin-coating speed of 7,000 rpm was used. At first, to apply the IGZO TFTs for pH sensing, (3-aminopropyl)triethoxysilane (APTES) solution was prepared to silanize the surface of IGZO TFTs. APTES reduces noise level of signal and improves the sensitivity of the sensors^28,29^. The variation in surface charge state of the APTES-treated devices according to the pH is illustrated in Fig. 3a. With many H^+^ ions present, that is, in the low pH environment, the pH-sensitive –NH_2_ groups protonate to –NH_3_^+^, and the surface becomes a positive charge^29,30^. On the contrary, in the high pH environment (low H^+^ ion concentration), the –OH group on the surface is deprotonated to –O^−^, and the surface becomes a negative charge. Variations in the surface charge caused by pH variations induce a modulation of electrical characteristics of the devices. In this experiment, a liquid-gate sensing was conducted to measure the electrical characteristics of the devices by defining the area that contacts with the solution using PDMS wells^31^. The electrical characteristics of the film- and wire-type IGZO TFTs exposed to various pH solutions are shown in Fig. 3b,c. In both film- and wire-type devices, drain current increased with increasing pH. This result is due to the surface charge of IGZO becoming positive by protonation of aminosilane groups in high pH environment, and these charges act as positive gate bias^29,30^. As shown in the inset of Fig. 3b,c, the sensitivities of the film- and wire-type devices were 27.6 and 45.4 mV/pH, respectively. The wire-type sensors have larger surface area compared with the film-type ones, and this large surface area resulted in superior sensing characteristics to the change of pH^20^. Figure 3d,e shows the representative response characteristics of the film- and wire-type sensors at pH that ranges of 9 to 5. Both film- and wire-type sensors showed linear characteristics with respect to the pH change^28,29^, and the variations in normalized current of the wire-type sensor were larger than those of the film-type one.

Additionally, a glucose sensor was prepared by immobilizing glucose oxidase after surface treatment with glutaraldehyde, which is a linker for bonding glucose oxidase with aminosilane groups. Glucose solutions with various concentrations were prepared by dissolving D-glucose in 1x phosphate buffered saline (PBS) and sensing was performed in the same manner as the pH sensing using glucose solutions. When glucose is exposed to glucose oxidase, an enzymatic oxidation reaction occurs as follows^32,33^:

$${\rm{D}} \mbox{-} {\rm{glucose}}+{{\rm{O}}}_{2}+{{\rm{H}}}_{{\rm{2}}}{\rm{O}}\,\mathop{\longrightarrow }\limits^{{\rm{glucose}}\,{\rm{oxidase}}}\,{\rm{gluconate}}+{{\rm{H}}}^{+}+{{\rm{H}}}_{2}{{\rm{O}}}_{2}$$

**Figure 4:**
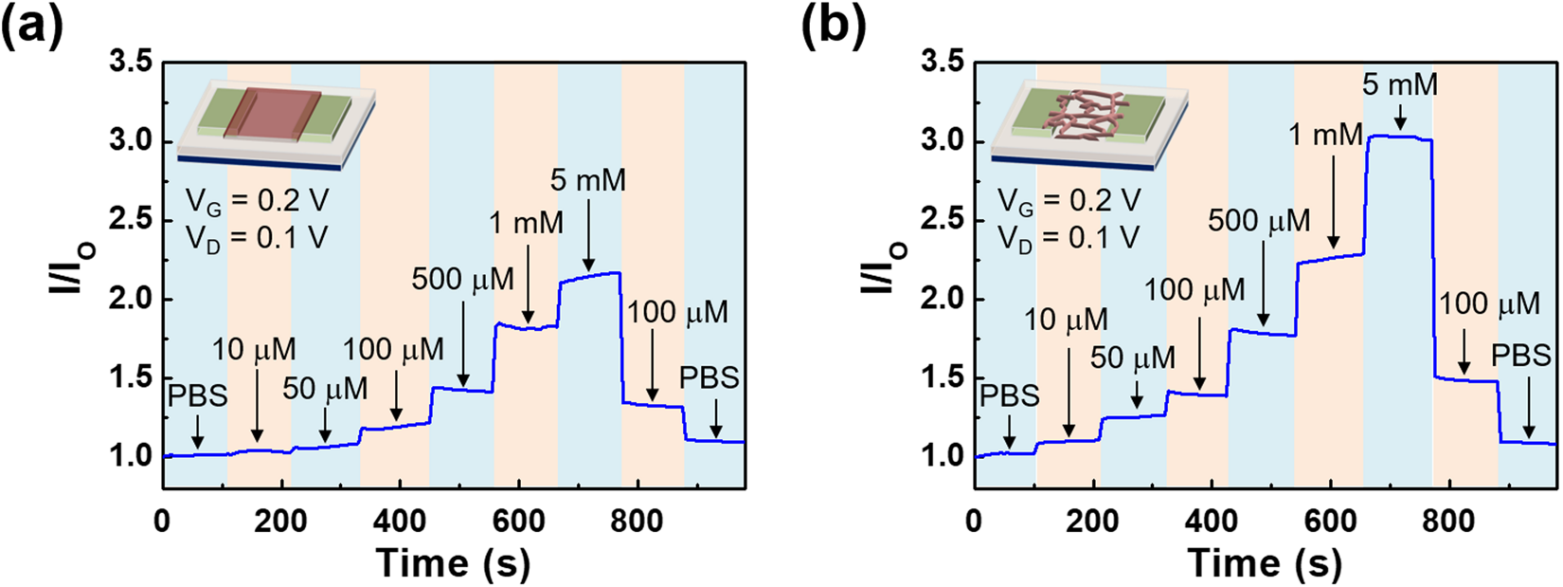
Real-time response of the drain current of (**a**) film-type and (**b**) wire-type IGZO sensors for the glucose solution with various concentrations.

The amount of generated H^+^ ions is varied with the concentration of the glucose, and the variation of H^+^ ion concentration influences the pH of the solution^34^. These variations of pH resulted in the modulation of electrical characteristics of sensors. Figure 4 shows the real-time detection of drain current with various concentration of glucose solution. Same as the pH sensing results, the wire-type device exhibited more changes in electrical characteristics, and such result was attributed to the large surface area of the wire-type sensors compared with film-type ones. In addition, to validate the selectivity of the glucose sensor, an additional experiment was conducted to verify whether the device characteristics were changed by Na^+^ ion. Figure S3 shows the current change of wire-type sensor according to the 1x PBS solution with various NaCl concentration. There is little change to the current under 1x PBS solution with additional 10 mM NaCl, and good selectivity against the Na^+^ ions of the fabricated sensor was verified.

**Figure 5:**
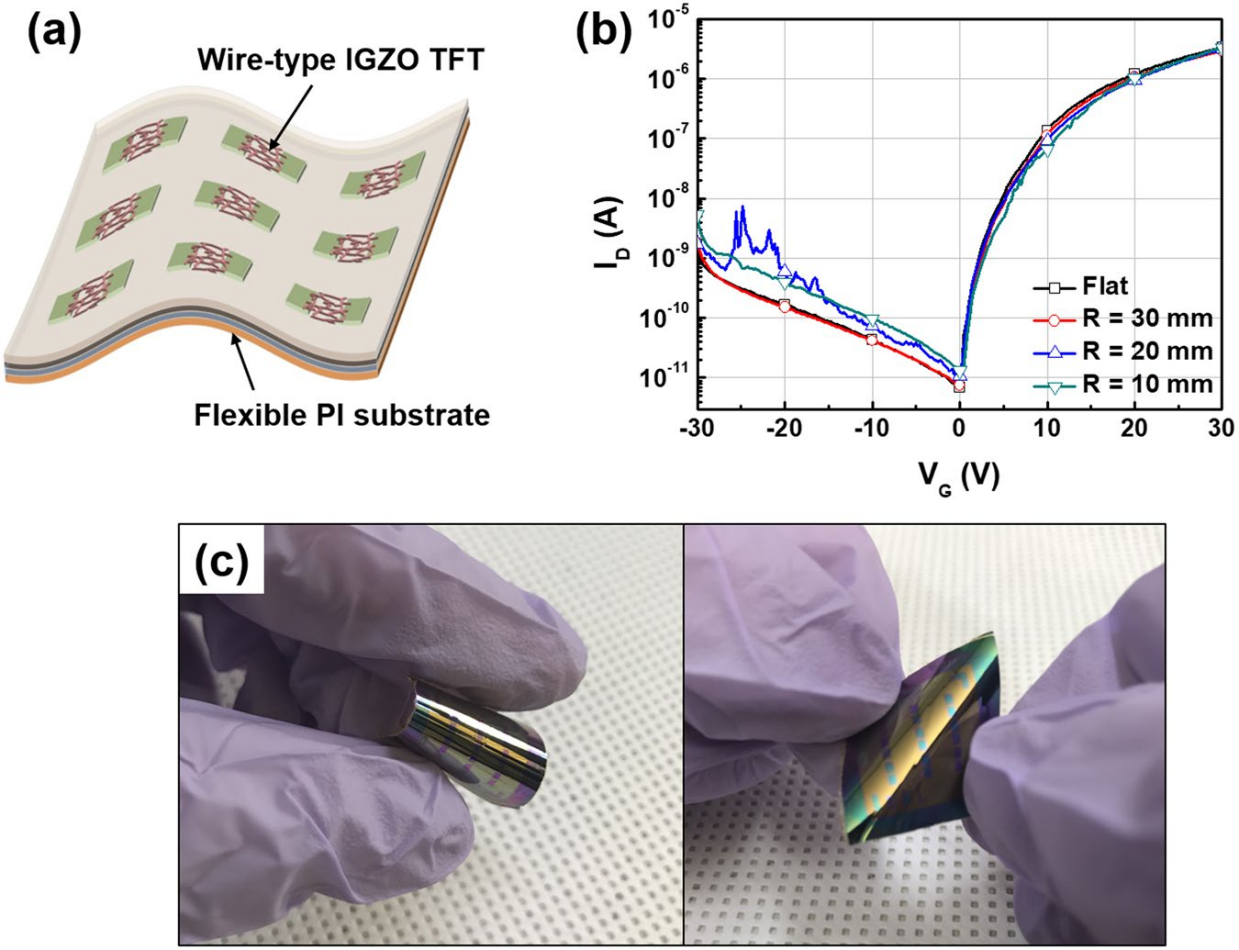
(**a**) Schematic diagram of wire-type IGZO TFTs on a flexible substrate. (**b**) Transfer characteristics of flexible wire-type IGZO TFTs measured in the bending state at various radii and (**c**) photograph of flexible wire-type IGZO TFTs.

Finally, we fabricated wire-type IGZO TFTs on a flexible substrate. A schematic diagram and the electrical characteristics of flexible TFTs are shown in Fig. 5. The flexible TFTs exhibited stable electrical characteristics even at a bending radius of 10 mm (see Fig. 5b). This result come from the more resistive characteristics of the nanostructure under mechanical stresses than the conventional film structure due to the preventing the extension of cracks^35–37^. Therefore, wire-type devices showed highly robust characteristics against the mechanical stress and can be used in flexible applications.

## Conclusion

We successfully fabricated the wire-type IGZO TFTs using a cracked template based on a lift-off process. The cracked template was readily formed by coating colloidal silica onto the substrate followed by drying; no additional treatment was required and the template was easily removed via ultrasonication. The width and density of the wires which lead to the variation in electrical characteristics of wire-type IGZO TFTs were controlled by modulating the spin-coating speed of the template solution or by stacking additional layers. Through surface modification, the fabricated wire-type devices were applied to pH and glucose sensors. The wire-type sensors showed higher sensitivity compared with the conventional film-type sensors in both pH and glucose sensing. Since the surface area of wire-type sensor is larger than that of film-type sensor, the sensitivity improvement of wire-type sensor was achieved. Wire-type TFTs were fabricated onto a flexible substrate using the same process; the electrical characteristics were stable even when mechanical stress was applied. Our fabrication method can be used not only to prepare ultrasensitive flexible sensors but also when interconnected wires are required; the method is simple and cost-effective.

## Methods

### Device Fabrication

Two types of TFTs, rigid and flexible types were fabricated in this study. All TFTs were of the bottom-gate bottom-contact structure. A heavily doped p-type Si wafer with a 120-nm-thick thermal oxide layer was used as the substrate when fabricating rigid-type transistors. After cleaning the substrate, a 200-nm-thick indium tin oxide (ITO) layer (the S/D electrode) was deposited by radiofrequency (RF) magnetron sputtering using a shadow mask. To make a hydrophilic surface, the substrate was irradiated with deep ultraviolet (UV) light (185 nm and 254 nm) and spin-coated with colloidal silica. After drying in air at room temperature for 5 min, a 40-nm-thick IGZO layer was deposited via RF magnetron sputtering using a shadow mask. The template was removed by ultrasonication in water. Residual water was blown off using nitrogen gas, and all devices were annealed at 300 °C for 1 h. The geometrical width and length of the TFT channel were 1,000 and 150 μm, respectively.

For the fabrication of flexible type TFTs, a 20-μm-thick polyimide (PI) film was formed on carrier glass and SiN_x_ and SiO_2_ buffer layers deposited via plasma enhanced chemical vapor deposition (PECVD). A 60-nm-thick Mo layer (the gate electrode) was deposited via RF magnetron sputtering and a 200-nm-thick SiO_2_ layer (the gate insulator) was fabricated via PECVD. Subsequent fabrication processes (S/D electrode deposition, active layer formation, and annealing) were performed using the same methods as the fabrication of rigid TFTs.

### Surface functionalization for glucose sensing

For surface silanization, fabricated IGZO TFTs were immersed in an ethanol-based 5% APTES solution for 6 h and rinsed with ethanol. To conjugate a linker to the amino groups of APTES, APTES-treated IGZO TFTs were soaked in a solution of 2.5% glutaraldehyde in 1x PBS for 1 h. After cleaning the samples using 1x PBS solution, immobilization of glucose oxidase on the surface of IGZO TFTs was conducted by dropping the glucose oxidase solution (10 mg of glucose oxidase in 1 mg of 1x PBS) onto the IGZO TFTs. After 12 h, finally, the samples were rinsed using 1x PBS solution.

### Film and Device Characterization

Structural images were obtained using field-emission scanning electron microscopy (FE-SEM; JEOL-7800F) and OM (Olympus BX51). For FE-SEM, a thin Pt coating was applied to all samples to enhance visibility of images. The electrical characteristics of the fabricated devices were measured using an HP4156C semiconductor parameter analyzer in the dark at room temperature. μ_FE_ was calculated using the following equation:

$${\mu }_{FE}=(\frac{2}{{C}_{ox}})(\frac{L}{W})({G}_{\max }){}^{2},$$

where C_ox_, L/W, and G_max_ are the gate insulator capacitance per unit area, the ratio of length to width of the channel layer, and the maximum value of the gradient (G), which was calculated by differentiating the square root of the I_D_ by the gate voltage (V_G_):

$$G=\frac{\partial \sqrt{{I}_{D}}}{\partial {V}_{G}}.$$

## Electronic supplementary material

Supporting Information
