# Supplementary material for: Facile fabrication of wire-type indium gallium zinc oxide thin-film transistors applicable to ultrasensitive flexible sensors
Source: Sci Rep. 2018 Apr 3;8:5546. doi: 10.1038/s41598-018-23892-4 (PMC5882893; doi:10.1038/s41598-018-23892-4)
Supplement: Supplementary file 1 — Supporting Information [file 41598_2018_23892_MOESM1_ESM.docx]

**Supporting Information**

**Facile fabrication of wire-type indium gallium zinc oxide thin-film transistors applicable to ultrasensitive flexible sensors**

# Yeong-gyu Kim, Young Jun Tak, Hee Jun Kim, Won-Gi Kim, Hyukjoon Yoo, and Hyun Jae Kim*

School of Electrical and Electronic Engineering, Yonsei University, 50 Yonsei-ro, Seodaemun-gu, Seoul, 03722, Republic of Korea.

*Corresponding author: Hyun Jae Kim (e-mail: [hjk3@yonsei.ac.kr](mailto:hjk3@yonsei.ac.kr))


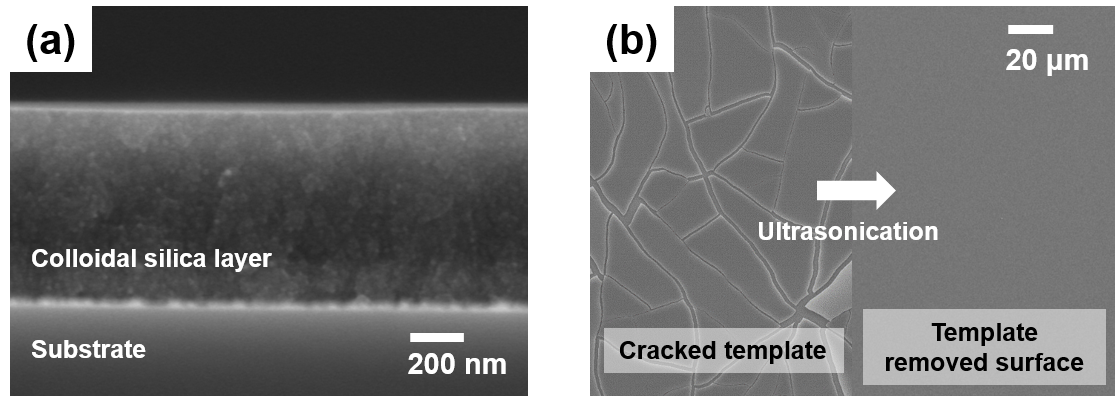


**Figure S1.** (**a**) Cross-sectional SEM image of fabricated template. (**b**) SEM image of the cracked template fabricated by colloidal silica (left) and the surface after removal of the cracked template using ultrasonication (right).


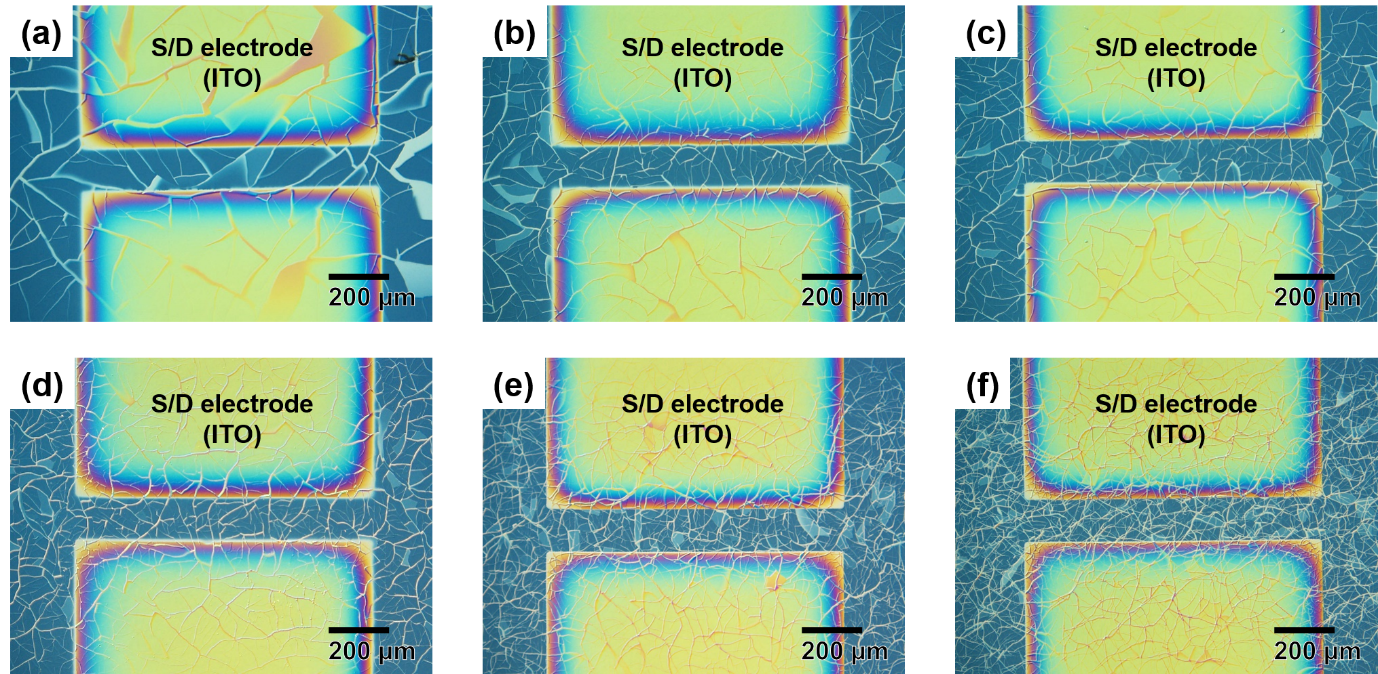


**Figure S2.** OM images of single-layered wire-type IGZO TFTs using different spin-coating speeds for template fabrication; (**a**) 1,000, (**b**) 3,000, (**c**) 5,000, and (**d**) 7,000 rpm. OM images of (**e**) double- and (**f**) triple-layered wire-type IGZO TFTs fabricated with spin-coating speed of 7,000 rpm for template fabrication.


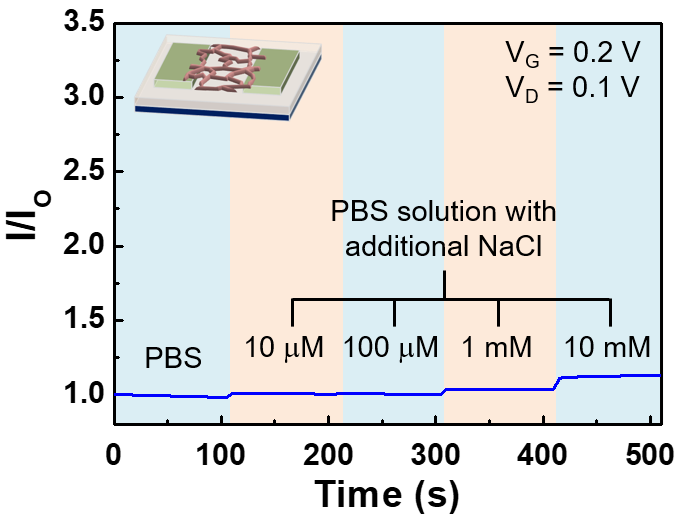


**Figure S3.** Representative response characteristics of the wire-type sensors with different NaCl concentration in PBS solution.
